# Is the auditory evoked P2 response a biomarker of learning?

**DOI:** 10.3389/fnsys.2014.00028

**Published:** 2014-02-20

**Authors:** Kelly L. Tremblay, Bernhard Ross, Kayo Inoue, Katrina McClannahan, Gregory Collet

**Affiliations:** ^1^Department of Speech and Hearing Sciences, University of WashingtonSeattle, WA, USA; ^2^Baycrest Centre, Rotman Research InstituteToronto, ON, Canada; ^3^Department of Medical Biophysics, University of TorontoToronto, ON, Canada; ^4^Department of Radiology, Integrated Brian Imaging Center, University of WashingtonSeattle, WA, USA; ^5^Life Sciences Department, Royal Military AcademyBrussels, Belgium; ^6^Unité de Recherche en Neurosciences Cognitives, Centre de Recherches en Cognition et Neurosciences Université Libre de BruxellesBrussels, Belgium

**Keywords:** auditory, training, ERP, P2, exposure, learning, rehabilitation, electrophysiology

## Abstract

Even though auditory training exercises for humans have been shown to improve certain perceptual skills of individuals with and without hearing loss, there is a lack of knowledge pertaining to which aspects of training are responsible for the perceptual gains, and which aspects of perception are changed. To better define how auditory training impacts brain and behavior, electroencephalography (EEG) and magnetoencephalography (MEG) have been used to determine the time course and coincidence of cortical modulations associated with different types of training. Here we focus on P1-N1-P2 auditory evoked responses (AEP), as there are consistent reports of gains in P2 amplitude following various types of auditory training experiences; including music and speech-sound training. The purpose of this experiment was to determine if the auditory evoked P2 response is a biomarker of learning. To do this, we taught native English speakers to identify a new pre-voiced temporal cue that is not used phonemically in the English language so that coinciding changes in evoked neural activity could be characterized. To differentiate possible effects of repeated stimulus exposure and a button-pushing task from learning itself, we examined modulations in brain activity in a group of participants who learned to identify the pre-voicing contrast and compared it to participants, matched in time, and stimulus exposure, that did not. The main finding was that the amplitude of the P2 auditory evoked response increased across repeated EEG sessions for all groups, regardless of any change in perceptual performance. What’s more, these effects are retained for months. Changes in P2 amplitude were attributed to changes in neural activity associated with the acquisition process and not the learned outcome itself. A further finding was the expression of a late negativity (LN) wave 600–900 ms post-stimulus onset, post-training exclusively for the group that learned to identify the pre-voiced contrast.

## Introduction

Long before the effects of auditory deprivation and stimulation on the brain were known, audiologists used auditory training exercises as a way to help people compensate for hearing loss (Carhart, 1960). The motivation for such exercises stemmed from the fact that adults and children with hearing loss often needed help in dealing with their speech perception deficits that remained after being fit with hearing aid amplification devices (Boothroyd, 2010). Some people reported training exercises to be helpful and others did not, so the use of auditory training exercises was questioned and slowly faded from clinical practice. By the year 2005, a mere 30% of audiology practices reported using auditory training type interventions in routine clinical practice (Kricos, 2006).

Advances in neuroscience reignited the interest in auditory training because of the plethora of research documenting the capacity of the human brain to change, depending on the type of sensory input or lack thereof. Here we focus on auditory perceptual training as a means of exploring the human capacity to learn so that brain plasticity can be optimized in ways that enhance the rehabilitation of people with hearing loss. Previous studies have shown that training-related changes in neural activity precede changes in auditory perception (Tremblay et al., 1998; Atienza et al., 2002) therefore, non-invasive physiological measures might provide an opportunity to monitor and optimize intervention efforts in people with different types of hearing loss.

Even though auditory training exercises in humans have been shown to improve certain perceptual skills of individuals with and without hearing loss (Boothroyd, 1997; Tremblay et al., 1997, 1998, 2001; Fu et al., 2004; Irvine and Wright, 2005; Sweetow and Sabes, 2006; Burk and Humes, 2007; Tremblay and Moore, 2012; Anderson et al., 2013; Chisolm et al., 2013; Sullivan et al., 2013), there is a lack of knowledge pertaining to which aspects of training are responsible for the perceptual gains, and which aspects of perception are changed (Amitay et al., 2006, 2013; Boothroyd, 2010; Henshaw and Ferguson, 2013; Jacoby and Ahissar, 2013). This lack of knowledge hinders the rehabilitation of people with hearing loss because individuals do not always respond as expected to the training program in which they participate. Even among normal hearing listeners, the effects of training can be highly heterogeneous. Without knowing which aspects of the training exercises are responsible for observed benefits, it is difficult to determine which components of the training paradigm are ineffective and what individual needs still require targeted intervention.

To better define how auditory training exercises impact brain and behavior, electroencephalography (EEG) and magnetoencephalography (MEG) have been used to determine the time course and coincidence of cortical and sub-cortical modulations in evoked activity associated with different types of auditory training (Tremblay et al., 1997, 2001, 2009, 2010; Brattico et al., 2003; Shahin et al., 2003; Bosnyak et al., 2004; Sheehan et al., 2005; Alain et al., 2010; Carcagno and Plack, 2011; Shahin, 2011; Anderson et al., 2013; Barrett et al., 2013). Here we focus on studies involving the P1-N1-P2 waves of the cortical auditory evoked response (AEP), as there are consistent reports of gains in P2 amplitude following various types of auditory training experiences; including music (Shahin et al., 2003; Kuriki et al., 2007; Seppänen et al., 2012; Kühnis et al., 2013) and speech-sound training. Despite converging evidence that increases in the amplitude of the P2 wave of the P1-N1-P2 complex coincides with improved perception, little is known about the functional meaning and neural generators of the auditory P2 response and whether or not it could serve as a biological marker of auditory learning. Our earlier studies show the center of activity for P2 to be in the anterior auditory cortex, but how this relates to learning is still unknown (Ross and Tremblay, 2009).

Speech sounds and acoustic elements thereof are represented in the neural activity patterns along the auditory pathway. One example is the representation of voice-onset time (VOT), as reflected through a sequence of onset responses recorded from primary auditory cortices in feline, primate, and human models (Eggermont, 1995; Steinschneider et al., 2005). Monotonic increases in VOT result in latency shifts and double onset responses involving the N1 peak of the P1-N1-P2 complex (Tremblay et al., 2003; Steinschneider et al., 2005). The N1 is often described to be an “exogenous” response, meaning that it is sensitive to physical characteristics of the sound used to evoke the response (see Picton, 2013 for a recent review). As an example, the N1 reflects the detection of acoustic changes; including, the onset of sound, and acoustic changes within an ongoing sound (such as a consonant-vowel transitions) (Ostroff et al., 1998; Wagner et al., 2013). The P1 wave is thought to reflect gating of auditory information to the auditory cortex (Alho et al., 1994) whereas the P2 may reflect auditory processing beyond sensation (Crowley and Colrain, 2004). It is for this reason; the P1-N1-P2 complex has been used to examine the neural representation of perceptually relevant temporal cues such as VOT.

In a series of past experiments, the effects of VOT training on the human P1-N1-P2 complex have also been studied (Tremblay et al., 2001, 2009, 2010; Sheehan et al., 2005; Alain et al., 2010). These experiments were used to determine if neural VOT codes could be altered through training. That is, could the perception of two within category VOT stimuli (e.g., identification and/or discrimination) that are perceived alike, and that evoke similar N1 peak latencies be altered with training? What’s more, if perception changes, does the neural representation of VOT, marked by the latency of N1, change?

The VOT training studies described earlier did not reveal modifications in the latency of the N1 response. Instead, P2 amplitudes increased following VOT training. Training-related enhancements in P2 turned out not to be specific to VOT or VOT training. Enhanced P2 amplitudes appeared after various types of sound exposures (Tremblay and Ross, 2007; Tremblay et al., 2001, 2009; Atienza et al., 2002; Bosnyak et al., 2004; Sheehan et al., 2005) including identification or discrimination training; for different types of stimuli including tones and speech sounds; presented in different types of event-related potentials (ERPs) contexts (homogenous block or oddball paradigm, monaurally or binaurally); over different time courses (1 day vs. 1 year); using EEG or MEG. The P2 effect is robust, can be reliably seen in individuals, and is retained for months following initial exposure (Tremblay et al., 2010). This phenomenon is not limited to the laboratory either; enhanced P2 amplitudes appear to reflect life-long learning such as musical training (Kuriki et al., 2006; Shahin, 2011).

Even though P2 amplitude gains have been reported to be physiological correlates of auditory learning, it is important to challenge this notion by recognizing that contributions of stimulus exposure, executive function, cognitive tasks, and memory are inherent in any auditory training paradigm. Any one or combination of these components, rather than learning itself, could be influencing P2 changes reported in the literature. In fact, our previous studies (Ross and Tremblay, 2009; Tremblay et al., 2010), and others (Sheehan et al., 2005) suggest that mere stimulus exposure, during EEG and MEG recording sessions and behavioral baseline testing, in the absence of training or changes in perceptual performance, contribute to enhanced P2 amplitude.

Expanding this program of research by including different experimental designs, while involving the same stimuli, enables us to identify converging evidence across the studies. Therefore, the purpose of this study was to determine whether or not P2 amplitude changes represent biologic markers of auditory learning. To do so required examining modulations in brain activity in a group of participants who learned the task and comparing it to participants, matched in time, task, and stimulus exposure, that did not learn. Modulations in P2 amplitude could be viewed as a biomarker of auditory learning if P2 amplitudes increased only for the group that learned the VOT contrast, but not in the other groups.

**Figure 1:**
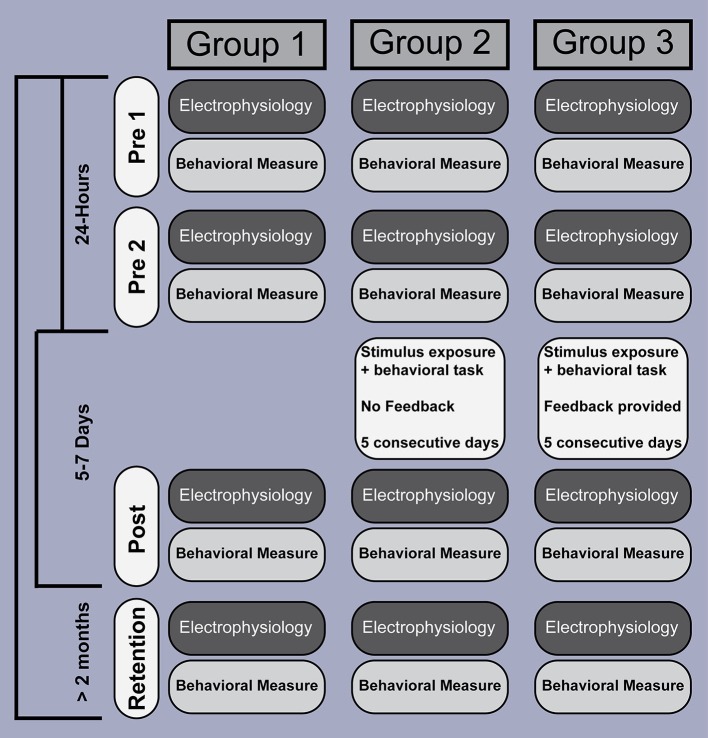
**Experiment design and time course**. EEG recording and behavioral testing was performed at similar points in time, across four sessions, and involved three groups. EEG data were acquired separate from the behavioral sessions. Whereas participants in Groups 2 and 3 were exposed to, and interacted with, the stimuli over a 5 day period between test sessions, Group 1 did not. The number of stimuli (amount of stimulus exposure) was identical across Groups 2 and 3, and participants were required to perform a similar task (click the mouse to advance to the next stimulus), but what differed between the two groups was the instructions and feedback. Participants in Group 3 received instructions and response feedback intended to improve their ability to correctly identify each of the two pre-voiced stimuli, but participants in Group 2 did not.

We therefore recorded behavioral responses and brain activity, elicited by stimuli differing in VOT, from three groups of participants, who were tested within similar time windows (Figure 1). The first group served as a control group without intervening listening or training experience, so that quantifiable modulations in brain activity could be related solely to the passage of time. The remaining two experimental groups (Groups 2 and 3) participated in listening tasks during a 5 day intervening period between pre 2 and post sessions. Both groups heard the same number of stimulus sounds during these intervening days, but the two groups differed in the type of task and feedback they received. One facilitated learning whereas the other did not. For example, members of Group 2 were asked to click a mouse button (to proceed to the next sound) after hearing each sound without receiving any feedback to facilitate learning the VOT contrast. Group 3 members were instructed how to label each sound (the two-alternative force-choice task) by clicking a mouse button, feedback about their performance followed so to facilitate learning. In doing so, we were able to examine brain-related changes in activity among a group that did and did not learn the VOT contrast. We also looked beyond a typical P1-N1-P2 time window (<200 ms in latency), to determine if VOT training modulates more endogenous, higher-level, aspects of sound processing.

## Materials and methods

### Participants

Thirty normal-hearing native-English speakers (18–39 years) were randomly assigned to one of three groups (10 in each group). Normal hearing was defined as pure tone thresholds ≤25 dB HL across frequencies between 250 and 8000 Hz. All participants were right handed and provided their written informed consent prior to participation. The Research Ethics Board of the University of Washington approved the study. Data from ten of these subjects (Group 1) were previously described in a publication that reported only the effects of repeated stimulus exposure (Tremblay et al., 2010).

### Stimuli

Two Klatt synthesized pre-voiced “ba” syllables, 180 ms in duration, were used in this experiment. They were the same stimuli used in a series of experiments designed to examine the neural encoding of VOT with training (see series of experiments by Tremblay et al., 1997 through 2010). Adult native English speakers consistently describe both pre-voiced stimuli as “ba” (McClaskey et al., 1983), but following training, they can learn to identify and label the −10 ms VOT stimulus as “ba” and the −20 ms VOT stimulus as “mba” (Tremblay et al., 1997).

### Behavioral tests

The ability to correctly identify the two stimuli was tested in four sessions for all groups within the same time frame (Figure 1). The first two tests were performed on 2 subsequent days, termed pre 1 and 2, and provided baseline performance scores. A post-training test was administered 5–7 days later and a retention test more than 2 months later. All groups were involved in the identification task, which was the same for all sessions. Participants were presented with randomized trials of the “mba” and “ba” stimuli. Twenty-five of the “mba” and 25 “ba” stimuli were presented in each session binaurally at a level of 76 dB SPL using insert earphones (Etymotic Research ER3a). The test was self-paced and a response, entered via a computer mouse, triggered the presentation of the next sound. Feedback was not provided in any test. The instructions to all participants were: “*You will hear some sounds and I want you to label the sounds as you hear them using the left button on the computer mouse. You will label the sounds based on two choices that will be displayed on the computer monitor. There is no right or wrong answer; it is simply your perception of what you hear*”. Two labels appeared on the computer screen as text: “mba” and “ba”.

### Behavioral training

Group 1 participated in the four-behavioral tests only and served as a control group for examining changes in perception and physiology, over the same time periods as Groups 2 and 3. Groups 2 and 3 participated in training sessions on 5 consecutive days, starting immediately following the pre 2 behavioral testing. Both groups heard four blocks of 50 randomized presentations of the “mba” and “ba” syllables, 25 of each on each day. Behavioral testing was self-paced and lasted approximately 20 min each day. Whereas the numbers of stimuli (amount of stimulus exposure) and the motor task of clicking the mouse were similar across the two groups, the instructions and feedback were different between groups. The task for Group 3 involved evaluating the stimulus they just heard, making a decision about what label they will assign to each sound, and then clicking the mouse to indicate which sound they heard. Group 3 also received feedback, which was intended to motivate participants to “correctly” label each sound.

Participants in Group 2 were instructed: “*You will hear some sounds. After each sound press the button on the screen to continue to the following sound*”. A button labeled “NEXT” was displayed on the computer screen to advance the task following each stimulus presentation.

Group 3 participants were instructed: “*Now, we’re going to help you label one sound /ba/ and one /mba/. You will be given feedback following each trial. If you select the correct label, it will turn green. If you do not select the correct label, the next trial will begin*”. Two text labels, “mba” and “ba”, were displayed on the computer screen.

### Electroencephalography (EEG) acquisition

EEG recordings and behavioral testing were completed in a sound-attenuated booth on 2 consecutive days (Session pre 1 and 2) 1 week following initial testing (post-training session) and 2 months to 1+ year following initial testing (retention session). Retention tests were staggered in time so changes in brain and behavior could be tracked over a large time window.

Similar to our previous experiments, stimuli were delivered monaurally via insert earphones to the right ear at 76 dB SPL; the same intensity was used for the behavioral tests. A passive EEG paradigm was used, meaning participants watched closed-captioned movies and were instructed to stay alert but no particular attention to the stimuli was requested. No behavioral task took place during EEG recordings. Four hundred presentations of the same type stimuli (“ba” or “mba”) were presented with an inter-stimulus interval of 1993 ms in a block. Following a 5 min break, a block of the other sound stimulus (“mba” or “ba”) was recorded. Stimulus order was counter-balanced across groups and test sessions. This particular ISI was used because our previous studies have shown that younger and older adults are differentially sensitive to stimulus presentation rates faster than 2 s and in future studies we wish to compare these data to those of older adults (Tremblay et al., 2004).

Continuous EEG signals were recorded from 59 electrodes using an elastic cap (Electro-cap International, Inc.) and a PC-based Neuroscan system (SCAN, ver. 4.3.3) with SynAmps2 amplifiers. The electrode montage followed an extended 10–20 system, reported in more detail in Tremblay et al. (2010). Four additional electrodes were placed on the inferior and outer canthus of each eye to monitor eye blink activity. EEG signals were referenced to the Cz electrode, analog bandpass-filtered between 0.15 and 100 Hz (12 dB/octave roll off), amplified with a gain of 500, and digitized at a sampling rate of 1000 Hz.

For offline analysis, an artifact correction procedure using BESA (5.2) was applied to reduce the effects of contamination from eye-blinks and ocular movements. Eye-blink artifacts were identified by a threshold criterion and corresponding waveforms were averaged to obtain a template of ocular artifacts. A principal component analysis of these averaged recordings provided a set of components that best explained the eye movements. The scalp projections of these components were then removed from the EEG signal to minimize ocular contamination.

In BESA the continuous EEG signal was parsed into stimulus onset related epochs of 1200 ms length, including a 200 ms pre-stimulus interval, which was used for baseline-correction. The signals were averaged for each stimulus condition and re-referenced to the average across all electrodes. Waveforms were low pass filtered at 32 Hz. The peak amplitudes and latencies of the N1 and P2 waves were measured as the signal maxima at electrode Cz in the latency intervals of ±50 ms around 100 ms and 200 ms for each participant, each stimulus type and each session.

## Results

### Behavioral data analysis

**Figure 2:**
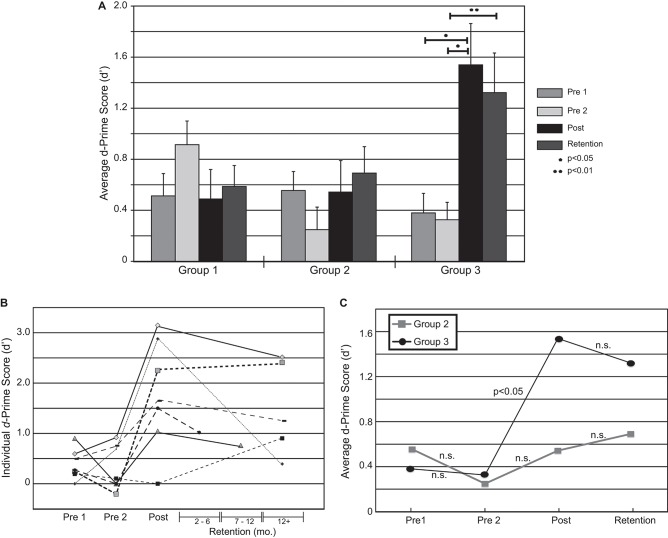
**Changes in behavioral performance over the time course of the experiment**. **(A)** Significant increases in performance were seen only for Group 3. Members of Group 3 participated in the identification task and received feedback. **(B)** Changes in d’ over time for 7 out of 10 individuals in Group 3 individuals who participated in the retention sessions. **(C)** A comparison in performance, over time, between Groups 2 and 3. Each group experienced the same number of trials and executed a button-pushing task, but Group 2 did not receive instructions or feedback designed to facilitate learning. No significant changes in performance were seen for Group 2.

To assess perceptual performance across groups, *d*-prime (*d*′) scores (Macmillan and Creelman, 1991) were computed for each participant from the rates of hits, misses, false alarms, and correct rejections for each behavioral test. A response was scored correct (hit) if the participant assigned the label “mba” to the −20 ms VOT stimulus. A correct rejection involved choosing the label “ba” for the −10 ms VOT stimulus. A split-plot 3 (fixed between groups; “Group”) × 4 (fixed within groups; “Session”) mixed model ANOVA was used to test the effects of “Group” and “Session” as well as their interaction on the *d*′-scores. *F-*statistics for the within-group effects and interactions were adjusted to control for Type I error due to significance of Mauchly’s test of sphericity. Follow-up pairwise comparisons were made using the Dunn-Sidak multiple comparisons procedure to control for Type I error. Figure 2A–C summarize the behavioral results. Significant improvement in identification performance was seen for Group 3 only, and was retained for as long as 1 year for some individuals.

### Performance evaluation—pre 1, pre 2 and post sessions

There was a significant main effect of “Session” on *d′-scores* (*F*_(2.33, 53.50)_ = 6.59, *p* < 0.01, *partial* *ω*^2^ = 0.13); as well as a “Group” × “Session” interaction (*F*_(4.65, 53.50)_ = 7.17, *p* < 0.001, partial *ω*^2^ = 0.28). Follow-up pairwise comparisons showed an increase in *d*′ between baseline (Pre 2) and the post-test for Group 3 only i.e., for those participants in the training who received performance feedback (*p-*values < 0.05).

### Performance retention

Figure 2B shows changes in *d*′-scores over time for Group 3. Three individuals were lost to attrition and were unavailable to return for retention testing. Analysis of *d*′-scores measured more than 2 months after the initial testing (Retention) revealed sustained improvements in performance for Group 3 (Figure 2A). Significant increases in *d*′-scores were seen between the baseline session (pre 2) and the post-training measures (*p* = 0.033), with significant differences between baseline and retention (*p* = 0.009) and no significant differences between post-training and retention measures (*p* = 0.697). An analysis of the *d*′-scores revealed that the improvements in performance, which was found between pre- and post-training measures persisted in the retention measure.

### Electroencephalography (EEG) analysis: auditory evoked responses

**Figure 3:**
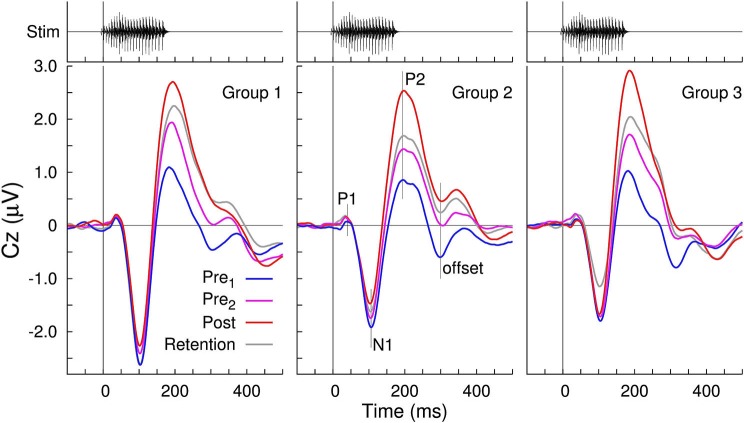
**Grand averaged voltages at the vertex electrode Cz in response to the −10 ms VOT stimulus “ba”**. Prominent N1 and P2 waves are visible in all time-series as well as the gradual increase in the P2 amplitude across the three sessions. Offset responses decrease across sessions, presumably due to the P2 amplitude growth.

**Figure 4:**
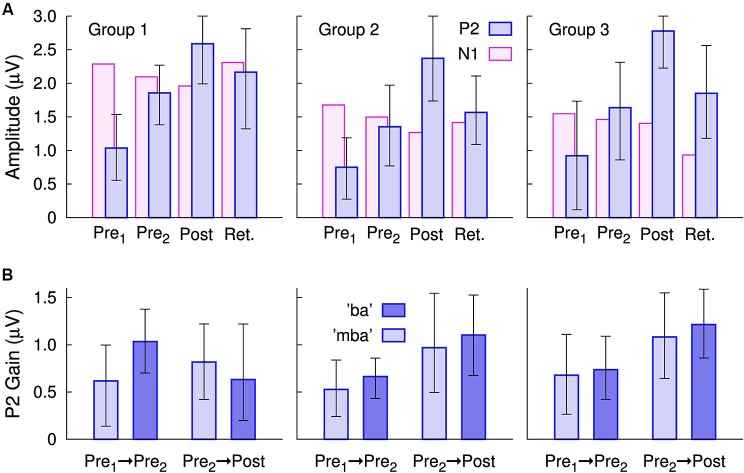
**Changes in the group mean P2 amplitudes, measured at electrode Cz**. **(A)** In all three groups, P2 amplitude gains were seen for the Pre 2, post-training, and even during the retention sessions. P2 amplitudes were still larger in the retention session than in the first session. By comparison, changes in the N1 amplitude were small. The error bars indicate the 95% confidence intervals for each group mean. **(B)** Gain in the P2 amplitudes between Pre 1 and Pre 2 sessions and between the Pre 2 and post-training sessions

To compare to our previously published studies, grand averaged evoked responses at electrode Cz are shown for the three groups and the four recording sessions in Figures 3, 4. All waveforms are in response to the −10 ms prevoiced “ba” stimulus and show prominent N1-P2 waves. The P1 wave is small. Although the response morphologies are quite different between groups as, for example, expressed in different ratios of the N1 and P2 amplitudes and variations at longer latencies beyond 300 ms, the effect of increasing P2 amplitudes between the first baseline recording and the post-training session is apparent in all three groups. Also, similar to our previous study (Tremblay et al., 2009), P2 amplitude measured across staggered retention sessions more than 2 months after the first recording, remained larger than the initially measured P2 amplitude. In contrast, changes in N1 amplitude over the time course of the experiment were small. Offset responses also appear to decrease over time, but we assume them to be driven by growth of P2.

The N1 amplitude showed smaller between-session changes than that of P2 (Figure 4A). N1 amplitude diminished over the time course of the first three recordings. A repeated measures ANOVA for the N1 amplitude revealed main effects of “Session” (*F*_(3,81)_ = 4.67, *p* = 0.0046) and “Stimulus” (*F*_(1,27)_ = 5.32, *p* = 0.029) and a “Session” × “Group” interaction (*F*_(6,81)_ = 3.11, *p* = 0.0087). When averaged across sessions, the stimulus effect appeared to be driven by the slightly larger “ba” amplitude for all three groups (mba: 1.63 µV and ba: 1.85 µV). The interaction diminished when considering the first three recordings only, suggesting it was mainly caused by the continuing N1 decrease in the retention session in Group 3 only. It should be kept in mind, that an N1 amplitude decrease means a positive voltage shift at the Cz electrode, which appeared in line with P2 amplitude increases, thus, a cross talk of the P2 changes has to be considered when interpreting the N1 changes. No significant changes in N1 latency were found for either stimulus, across sessions.

A repeated measures ANOVA on P2 amplitude with the between subjects factor “Group” (3 levels) and the within subjects factors “Session” (4 levels) and “Stimulus” (2 levels) revealed no main effect of “Group” (*F*_(2,27)_ = 0.5), but there were main effects of “Session” (*F*_(3,27)_ = 62.7, *p* < 0.0001) and of “Stimulus” (*F*_(2,27)_ = 13.6, *p* = 0.001). No “Group” × “Session” or “Group” × “Stimulus” interaction was significant. For the mean across groups, the P2 amplitude increased from 0.90 µV to 1.61 µV between the pre 1 and 2 baseline recordings, continued to increase to 2.59 µV in the post-training session, and decreased to 1.86 µV in the retention session. Compared to the first baseline recording, the P2 amplitude increased by 79% at the second baseline recording, by 187% at the post-training session, and retained larger than twice the initial amplitude after more than 2 months.

Gains in P2 amplitude between the pre-training sessions and between pre- and post-training sessions are illustrated in Figure 4B. An ANOVA revealed a main effect of “Session” (*F*_(1,27)_ = 4.9, *p* = 0.035) and a “Session” × “Group” interaction (*F*_(2,27)_ = 5.2, *p* = 0.030) because the P2 gain between pre- and post-training was larger than the P2 increase between the baseline sessions in Group 2 (*t*_(19)_ = 2.21, *p* = 0.040) and in Group 3 (*t*_(19)_ = 2.31, *p* = 0.035) but not in Group 1 (*t*_(19)_ = 0.3). There were no differences in the amount of P2 gain between Groups 2 and 3.

**Figure 5:**
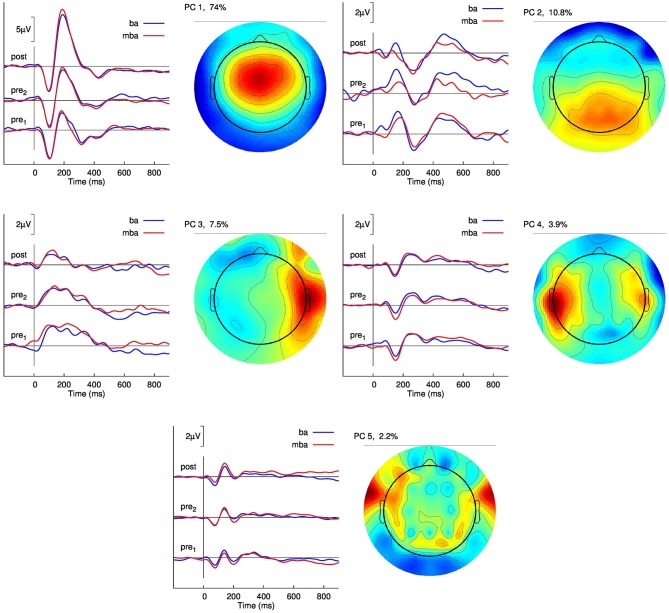
**Principal component analysis of the group averaged responses in the training group with feedback (Group 3).** The graphs show the time series of the five largest principal components (PC) for the two pre-training and the post-training recordings. The responses to the “ba” and “mba” stimuli are overlaid for comparison. The topographic map of the potential distribution across the head is shown right to the graphs of time series. The first PC explains 74% of the signal variance and shows the typical fronto-central maximum corresponding to two tangential dipole sources in left and right auditory cortices.

Results of a spatio-temporal principal component analysis on the evoked response waveforms observed in Group 3 are summarized in Figure 5 with the topographic distributions of the five largest components, which explain in total 98.4% of the variance, and the corresponding waveforms separately for the two baseline sessions and the post-training session. Overlaid are the responses to the “ba” and the “mba” stimuli. The aim of this analysis was to explore whether learning to identify the two stimuli would result in a different responses to “ba” and “mba”. Recognizing there are spatial precision limitations with EEG, we report the largest component, characterized by the N1-P2 waves, as being maximal at frontal midline electrodes, and the second largest component was predominant above the posterior parietal region. Smaller components were localized to left and right temporal and inferior frontal regions. Although the smallest component explained only 2.2% of the signal variance, the corresponding time series were clearly reproduced between sessions. Most importantly, no clear distinction between “ba” and “mba” responses became obvious. Accordingly, a formal multivariate test using PLS analysis showed a main effect of “Session” but no “Session” × “Stimulus” interaction. So far, the current data do not suggest that learning results in different cortical representation of the learned stimulus item beyond the statistical power of our analysis.

### Late negativity

**Figure 6:**
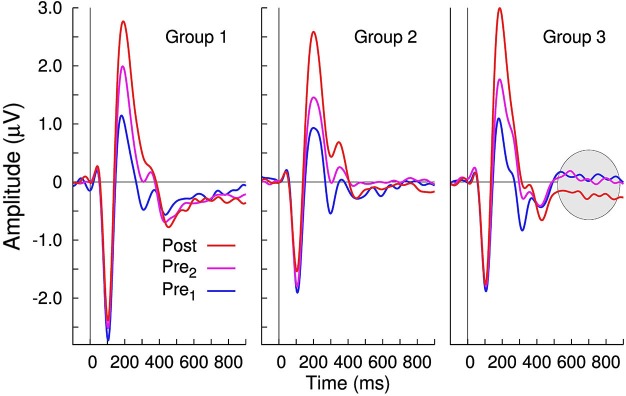
**Group averaged time series of the largest principal component of the evoked responses**. Whereas no between-session changes in the 600–900 ms latency interval are noticeable in Groups 1 and 2; in Group 3, there is an increased negativity following training.

Changes in evoked neural activity, in the 600–900 ms latency interval, were also observed during post- training and retention-sessions in the trained Group 3. Therefore, the mean amplitude in the 600–900 ms latency interval was measured and compared between groups and recording sessions. The repeated measures ANOVA for this late negativity (LN) revealed only a tendency toward significance for “Group” (*F*_(2,27)_ = 2.71, *p* = 0.085); however, a main effect of the within-subject factor “Session” (*F*_(2,54)_ = 6.92, *p* = 0.0021) and the “Session” × “Group” interaction (*F*_(4,54)_ = 3.47, *p* = 0.0135) was observed. Pairwise comparisons help to explain the interaction because between-session differences in the LN were significant in Group 3 only (Figure 6). In Group 3, the LN was larger after the training compared to the pre 1 session (*t*_(19)_ = 4.18, *p* < 0.0001) and compared to the pre 2 session (*t*_(19)_ = 3.62, *p* = 0.0018). Despite the significant training-related changes in the LN latency range for Group 3, the magnitude of perceptual change did not correlate with the amount of amplitude LN change (*R*^2^ = 0.07, *F* = 0.61, *p* = 0.46). Also, even though a visible LN can be seen in the retention data, the between subject variability was large (likely because of the staggered test times) and thus the retention effect was not significant.

## Discussion

There is a long history of using auditory training exercises as a part of auditory rehabilitation programs for people with and without hearing loss. One assumption is that listening training modifies the way sound is encoded and processed in the central auditory system, another is that listening exercises permit the person to make better use of existing neural codes. We still do not know what aspects of auditory training are responsible for perceptual gains (Boothroyd, 2010) and how coincident changes in neural activity relate to the auditory cue being trained. To address this issue, we compared training-related changes in perception and physiology, evoked by the same VOT stimuli, so brain and behavior relationships could be made. N1 and P2 latencies are consistently reported to be important neural correlates of VOT (Wagner et al., 2013); however, when people are trained to alter the perception of VOT, P2 amplitude, rather than N1 or P2 latencies are observed. Therefore, the purpose of this experiment was to determine if the auditory evoked P2 response is a biomarker of VOT learning.

### Is P2 a biomarker of learning?

The main finding was that P2 amplitude growths were observed for people who did and did not learn the novel VOT contrast. Based on these data, the most obvious conclusion is that P2 amplitude is not a biomarker of learning. This conclusion is reinforced by the growing body of evidence suggesting it is the elements of training (exposure, task execution) that contribute to P2 enhancements, and not the learned product of a goal-directed act. It would also explain why no study has been able to establish a one-to-one relationship between the magnitude of P2 change and the magnitude of perceptual change (Tremblay et al., 2001; Sheehan et al., 2005) and why enhancements appear to generalize to other stimuli exposed to but not necessarily learned (Tremblay et al., 2009). However, it is also possible that the large training related changes in P2 might overlay and obscure smaller effects of learning; or reflect other related processes not measured here. Therefore, to entirely dismiss a relationship between P2 and auditory learning would be to ignore converging evidence, from multiple laboratories, linking enhanced neuroplastic P2 activity to multiple forms of learned behaviors. When learning to discriminate the rate of frequency modulation in tones, for example, differences in performance gain related to different learning strategies, and were reported be reflected in P2 amplitude increases (Orduña et al., 2012). In a study of pitch discrimination training, absolute P2 amplitude correlated with reaction time (Tong et al., 2009). Also, long-term experience in musicianship and effects of auditory training in musicians were expressed in larger P2 amplitudes and amplitude increments compared to non-musicians (Seppänen et al., 2012). Collectively, there is a growing body of literature linking enhanced P2 amplitudes to auditory learning that it makes it difficult to entirely reject some type of brain-behavior relationship. We therefore put forth an alternative hypothesis; changes in P2 amplitude reflect neural activity associated with the acquisition process, but not the learned outcome itself. What neural mechanisms are associated with the process, and driving modulations in P2 activity, still need to be defined.

Based on source modeling we can assume some degree of auditory cortex involvement. Ross and Tremblay (2009) showed N1 and P2 to originate from different anatomical structures that likely serve different functions. N1 sources lay in the posterior part of auditory cortex, the planum temporale, whereas the center of activity for P2 lay in anterior auditory cortex, the lateral part of Heschl’s gyrus. P2 sources have also been identified in planum temporale, Brodmann’s area 22, and auditory association cortices (Crowley and Colrain, 2004). Whereas the P2 increase exhibits a neuroplastic nature, with enhanced activity becoming evident only after a period of sleep (Atienza et al., 2002; Ross and Tremblay, 2009; Zhu, 2010) and persisting for months; decreases in N1 amplitude occur within an experimental session and return to baseline in subsequent recordings (Ross and Tremblay, 2009). This type of N1 behavior pattern is more in line with habituation and less so with the types of learning–related N1 changes exhibited during active EEG recordings where modulations in brain activity are recorded while the participant is attending and executing the training task (Alain et al., 2010). Then again, habituation is sometimes termed “non-associative learning” and may be facilitating the P2 effects reported here (Rankin et al., 2009). N1 suppression mechanisms may also help consolidation, resulting in an increase of P2 between sessions.

The stimuli and passive recording paradigms used in our original VOT studies were designed to determine if neural codes reflecting VOT, and reflected by the N1, could be altered through training. If so, these far-field AEP recordings could be used clinically to assess the temporal resolution and rehabilitation of populations with suspected temporal processing disorders. The passive EEG recording paradigm is ideal for difficult to test populations and avoids potential confounds that can interfere with perceptual performance. Moreover, the stimulus block design was designed with future clinical applications in mind as these types of recording paradigms are within the capacity, and similar to electrophysiological procedures, used in audiology clinics today. However, to date, using this approach, no evidence of significant N1 latency shifts, reflecting perceptual changes in VOT, over time, have been reported. One possibility is that N1 latencies do not reflect subtle differences in *pre*-voicing. Another is that mechanisms underlying N1 are resistant to training (Wagner et al., 2013), or changes in synchronous activity are so modest that they cannot be detected using far-field recordings in humans. However, there is some evidence that N1 (and some subcomponents) can be modified with training but these were all observed as amplitude rather than latency changes (Menning et al., 2000; Brattico et al., 2003; Bosnyak et al., 2004). An exception is Reinke et al. (2003) who reported decreased N1 and P2 latencies, as well as enhanced P2 amplitudes following training, but these latency changes were recorded using an active EEG task while listeners partook in a vowel segregation-training task. This means, attention, auditory and visual sensory processing, memory and executive function could have contributed to the observed latency changes. Thus, it is difficult to differentiate sensory vs. cognitive (top-down) contributions to learning, as well as the various types of top-down contributors.

The P1-N1-P2 responses recorded here were acquired in a passive way and as such are described as being mainly exogenous in nature, meaning they are highly dependent on the physical properties of the stimulus used to evoke it. However, these AEPs can be endogenous, and modulated by attention in certain circumstances (Hillyard et al., 1973; Woldorff and Hillyard, 1991; Woods, 1995). This point is important when considering potential contributors to enhanced P2 activity. In our design, participants heard stimuli during the AEP sessions and during each perceptual training and testing task. They saw visual instructions and text response options. In all instances, auditory and visual input tapped into memory sources because sessions were repeated on different days. So, as described by Tremblay et al. (2001) and others, it is possible that some of the training-related physiological changes reported here might reflect other top-down modulatory influences that are activated during AEP recordings as well as focused listening tasks. What’s more, the P2 effects might not even be auditory specific. Similar to the auditory evoked P2, the visually evoked P2 is modulated by attention, language context information, and memory and repetition effects. It is also considered to be part of cognitive matching system that compares sensory inputs with stored memory (Luck and Hillyard, 1994; Freunberger et al., 2007). Therefore, although our source modeling studies (Ross and Tremblay, 2009) showed involvement of primary and association cortical areas, we have not yet ruled out multisensory interactions from contributing to our results. Until future experiments are designed to disentangle the various multi-sensory top-down contributing components such as: attention, memory, and executive function, we are left to speculate about neural mechanisms, and their contributions to the results reported here.

One possibility worth exploring in future studies is the concept of object representation (Näätänen and Winkler, 1999; Ross et al., 2013). If we view N1 and P2 as reflecting synchronous evoked auditory involved in the early stages of perceptual learning, where the neural representation of the sensory input takes place, we could speculate that the P2 indicates memory updating, and consolidation, where the two similar sounding (“ba” and “mba”) stimuli, are stored in a buffer. This phase could be passive, not requiring engagement of the participants, which would explain enhanced P2 activity from session to session in the absence of training. With directions and feedback, it would become possible to separate this sensory information into two objects “mba” and “ba”. Within this framework, we suggest that P2 plays a role in stimulus familiarization and auditory object representation; critical processes for successful perception. The second phase of learning is likely mediated by top-down processes and probably involves many interactive aspects involving attention, motivation, reinforcement etc. Whereas the first stage applies to the neural detection of sound, the second stage reflects how the brain makes use of the sound. To better understand later stages in sound processing, we expanded our prior analyses to determine if auditory training, and its components, result in recordable modulations in brain activity—later in time. As seen in previous studies (Tremblay et al., 2009) there might be experience-related changes occurring outside the P2 latency region that are visible in different scalp locations.

### Late negativity (LN)

A previously unreported finding was the presence of the LN in the post-training session, for the group that learned to identify the pre-voiced contrast. It appears to a lesser degree in the retention data as well, but brain-and-behavior scores do not correlate with each other. Like the P2, the magnitude of LN change does not predict a person’s perceptual change score. So what does the LN reflect?

It is well established that distinct forms of cognitive control are associated with unique patterns of activation over a distributed network of regions. These networks can include the dorsolateral prefrontal cortex (DLPFC), ventrolateral prefrontal cortex (VLPFC), supplementary and pre-supplementary motor areas, the anterior cingulate cortex (ACC), superior and inferior aspects of the posterior parietal cortex (Corbetta and Shulman, 2002; Cole and Schneider, 2007). What’s more, many aspects of cognitive control have been shown to manifest themselves as negativities in ERP recordings (e.g., N2, Nd, MMN, N400, Late Difference Negativity (LDN) and Error Related Negativity (ERN)). However, these types of negativities are typically recorded when the task involved attention switching, or other complex stimulus paradigms like an oddball paradigm, or often require active participation during the EEG recording. In the present experiment, participants were not engaged in a purposeful attention task and the stimuli were presented as a homogenous train of equiprobable events with no salient deviant stimuli. Thus, our use of the term LN is descriptive and does not neatly fit a well-characterized ERP profile. If left to speculate, we hypothesize that members of Group 3 learned to identify subtle acoustic cues that separated the two pre-voiced stimuli prior to the final ERP session. It is possible then that the training sessions drew greater attention to the stimuli as being separate objects. At the time of post-training EEG sessions, these two stimuli were automatically recognized as two separate auditory objects, but members of Group 3 were the only ones who were taught to attach each object to a perceptual label.

## Summary and conclusions

The purpose of this study was to determine if enhanced auditory evoked P2 activity is a biomarker of learning. The question is relevant to the study of auditory rehabilitation in that neurophysiological correlates of auditory training are needed to better understand the mechanisms of action presumed to be involved when using training as an intervention approach for people with and without hearing loss. This study showed increases in P2 AEP amplitude following exposure to auditory stimuli as well as the participation in tasks (with and without feedback). Enhanced P2 amplitudes were seen regardless of any change in perceptual performance and therefore not interpreted to be a biomarker of learning. Instead, modulations in P2 amplitude were attributed to changes in neural activity associated with the acquisition process and not the learned outcome itself. A process that is robust enough to be retained for months. A further finding was the expression of a LN wave 600–900 ms post-stimulus onset, in the post-training session, exclusively for the group that learned to identify the pre-voiced contrast. Collectively, we conclude that being exposed to and interacting with sound, alters the way those sounds are represented in the brain and these changes in neural activity are part of the learning process. Consistent with our earlier findings (Tremblay et al., 1998, 2009), changes in neural activity appear to precede changes in auditory perception and are retained for months. The application of this information to the assessment and rehabilitation of people with hearing loss and other communication-based disorders will depend on future studies aimed at disentangling multi modal bottom-up and top-down neural mechanisms contributing to changes in the N1, P2 and LN. However, a final take home point is that research directed at identifying neural mechanisms related to training and learning should take into consideration the contribution of repeated stimulus exposure as well as other possible coincident contributors to reported physiological changes.

## Author contributions

All authors contributed substantially to the concept/design of the work; the interpretation of data; draft reviews including revisions, and approved the final version to be published. Collectively we are accountable for all aspects of the work, including accuracy and integrity. Additionally, Katrina McClannahan and Gregory Collet contributed to data collection.

## Conflict of interest statement

The authors declare that the research was conducted in the absence of any commercial or financial relationships that could be construed as a potential conflict of interest.
